# Endowing textiles with self-repairing ability through the fabrication of composites with a bacterial biofilm

**DOI:** 10.1038/s41598-023-38501-2

**Published:** 2023-07-14

**Authors:** Anqi Cai, Zahra Abdali, Dalia Jane Saldanha, Masoud Aminzare, Noémie-Manuelle Dorval Courchesne

**Affiliations:** Department of Chemical Engineering, McGill University, 3610 University Street, Montreal, QC H3A 0C5 Canada

**Keywords:** Biomaterials - proteins, Biomaterials - proteins

## Abstract

To address the increasing environmental footprint of the fast-growing textile industry, self-repairing textile composites have been developed to allow torn or damaged textiles to restore their morphological, mechanical, and functional features. A sustainable way to create these textile composites is to introduce a coating material that is biologically derived, biodegradable, and can be produced through scalable processes. Here, we fabricated self-repairing textile composites by integrating the biofilms of *Escherichia coli* (*E. coli*) bacteria into conventional knitted textiles. The major structural protein component in *E. coli* biofilm is a matrix of curli fibers, which has demonstrated extraordinary abilities to self-assemble into mechanically strong macroscopic structures and self-heal upon contact with water. We demonstrated the integration of biofilm through three simple, fast, and scalable methods: adsorption, doctor blading, and vacuum filtration. We confirmed that the composites were breathable and mechanically strong after the integration, with improved Young’s moduli or elongation at break depending on the fabrication method used. Through patching and welding, we showed that after rehydration, the composites made with all three methods effectively healed centimeter-scale defects. Upon observing that the biofilm strongly attached to the textiles by covering the extruding textile fibers from the self-repair failures, we proposed that the strength of the self-repairs relied on both the biofilm’s cohesion and the biofilm-textile adhesion. Considering that curli fibers are genetically-tunable, the fabrication of self-repairing curli-expressing biofilm-textile composites opens new venues for industrially manufacturing affordable, durable, and sustainable functional textiles.

## Introduction

The textile industry constitutes an important sector of the global economy. The demand for fast fashion has resulted in increased production of clothing, footwear, and household fabrics, leaving behind an indelible environmental footprint^1^. Specifically, textile production involves substantial use of land, water, and raw materials, part of which are synthetic fibers sourced from fossil fuels. Textile consumption also raises environmental concerns due to improper disposal of unwanted or damaged clothes^2^. To tackle these problems, improving the durability and repairability of clothes is crucial, because functional damages, such as wear and tear, often account for consumers prematurely discarding their clothes^3^.

Recently, researchers have proposed some creative solutions to enable conventional textiles to self-repair permanent damages and regain lost morphological and functional characteristics. In particular, they employed self-healing polymers as textile coatings to create textile hybrids that can recover damages in response to environmental changes^4^. Although various physical coatings and chemical surface modification methods have been developed, most self-repair mechanisms reported only recover superficial, micro-sized damages or damages on individual fibers^4,5^. In addition, the industrial applicability of these self-repair mechanisms remains uncertain due to the complexity of the required chemistries and techniques, the biotoxicity and bioaccumulation of certain coating chemistries^6^, and the safety and pollution concerns associated with using organic solvents^7,8^.

Modifying conventional textiles with self-healing biological materials presents an alternative solution to repair textiles, because these materials are biocompatible, biodegradable, and have the potential for genetic customization. For example, structural proteins that self-assemble into higher-order structures in nature and exhibit outstanding mechanical strength are usually used for the mechanical improvement of composite materials and devices^9^. Through synthetic biology, structural proteins, such as Squid Ring Teeth proteins and silk proteins, have been produced by bacteria and applied in the fabrication of self-repairing textiles^10,11^. In these textile hybrids, novel functions, including biomolecule incorporation^10^ and environment-responsive self-regeneration^11^, have also been enabled. However, recombinant protein production and purification at an industrial scale can be costly^12^, hampering the practical use of these proteins in textile repair.

Recently, biofilms that are naturally self-produced by bacteria and self-assembled into higher-order structures have been exploited as biomaterials and incorporated into textile composites. Biofilms are co-adhered microbial cells enclosed in extracellular polymer matrices and attached to surfaces^13^. This material has the potential to be industrially produced in inexpensive, scalable, and non-polluting processes, hence complying with green and sustainable production. Textiles’ roughened surfaces and large pore volumes facilitate the development and adhesion of biofilm^14^, which inspired the construction of biofilm-textile hybrids^13,15,16^. Biofilm formation protects the biological activities of cells from environmental stresses, rendering the biofilm-textile composites self-regeneratable and environment-adaptable^15,16^.

Within the biofilms of enteric bacteria like *Escherichia coli* (*E. coli*), amyloid curli fibers are expressed as the major proteinaceous extracellular structural component and mediate cell–cell and cell-surface interactions^17,18^. The major subunit of curli fibers, CsgA, is secreted outside the cell membrane and nucleated and anchored to the cell wall^19^. By fusing CsgA with non-native peptides on the genetic level, a variety of functional biofilms have been developed for applications such as metal surface adhesion, inorganic nanoparticles templating^20^, enzyme immobilization^21^, light-inducible biomineralization^22^, and therapies for intestine inflammation and gut healing^23,24^. Owing to their ordered β-sheet-rich structures, amyloids like CsgA exhibit remarkable mechanical properties and stability with Young’s moduli ranging from 3 to 20 GPa^25^ and resistance to chemical and biological degradation^26^. Of particular interest, thin films made with curli fibers have demonstrated self-healing abilities through healing scratches/cuts and restoring functional features after rehydration with water^27,28^. Upon addition of water, curli fibers swell due to hydrogelation with increasing fibril entanglement and side chain-mediated non-covalent interactions between the fibrils, thus forming polymer networks^29–31^. Also, the recovery of multiple non-covalent interactions and supramolecular hydrogen-bonding networks can drive the self-assembly of curli fibers, promoting structural stabilization and self-healing^32–34^.

Inspired by the tunability, mechanical strength, and self-healing ability of curli fibers expressed in biofilms, we integrated curli biofilm into conventional knitted cotton textiles to fabricate self-repairing textile composites. We used three integration methods—adsorption, doctor blading, and vacuum filtration—to cover cotton textiles with biofilm and tuned biofilm density per textile surface area. While retaining breathability, the textile composites have enhanced mechanical properties depending on the integration method. We showed that the textile composites can effectively repair centimeter-sized cuts by patching and welding in the presence of water, and the strength of repaired composites was determined by the cohesion of biofilm and biofilm-textile adhesion. This work opens the opportunity for the scalable fabrication of sustainable and smart textile composites with novel functionalities and improved lifespan.

## Results and discussion

### Fabrication of biofilm-textile composites

**Figure 1:**
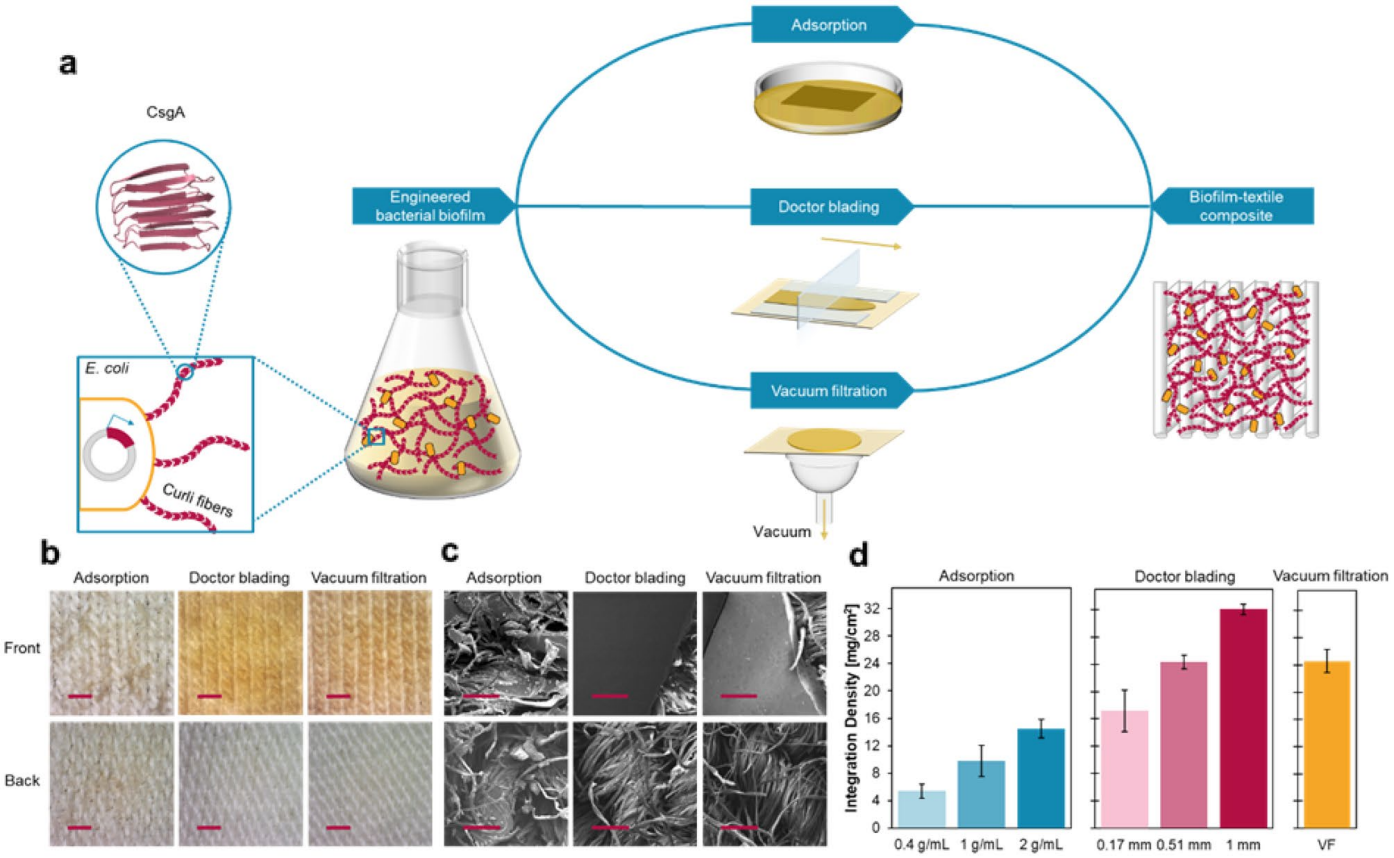
Fabrication of biofilm-textile composites. (**a**) A scheme of the three methods to integrate biofilm formed with curli fibers, amyloids assembled from CsgA (structure prepared by PyMOL and previously derived computationally^40^), into textiles. (**b**) Optical microscope images of the front and back sides of the biofilm-textile composites AS-2 g/mL, DB-1 mm, and VF. The scale bars are 1 cm. (**c**) SEM images of the front and back sides of the samples shown in (**b**), demonstrating the distribution and coating of biofilm on textile surfaces. The scale bars are 200 µm. (**d**) Bar plot of the integration density calculated for the composites prepared using the three methods. The bars represent mean values, and the error bars are standard deviations.

First, we aimed to fabricate composites with curli-expressing *E. coli* biofilms and cotton/spandex jersey knitted fabrics (Fig. S1). To prepare the composites, we used bacterial cultures where amyloid curli fibers were expressed and secreted in the medium (verified with a Congo Red binding assay, Fig. S2). We developed three composite fabrication methods that are fast, simple, and scalable to distribute biofilm on the textile surface differently and to endow the composites with different characteristics potentially suitable for a range of applications: method (1) depositing biofilm throughout the textile by incubating the textile in biofilm solutions of various concentrations (adsorption of the biofilm onto the surface of the textile fibers), method (2) applying a uniform biofilm layer with controlled thicknesses onto the textile (doctor blading), and method (3) filtering a concentrated biofilm solution through the porous textile under vacuum (vacuum filtration) (Fig. 1a). By altering the quantity of biofilm incorporated, we also aimed to allow for further adjustment of the composites’ characteristics.

We sought to compare the surface morphology of the composites made with the three methods. Adsorption resembles the dip coating technique commonly used to introduce compounds from a homogeneous solution with varying concentrations to modify textile surfaces^35^. We immersed the textiles overnight in biofilm solutions of 0.4 g/mL, 1 g/mL, and 2 g/mL (AS-0.4 g/mL, AS-1 g/mL, AS-2 g/mL) to provide a wet environment for biofilm infiltration into the textile matrix and sufficient time suitable for the irreversible adsorption of biofilm on both surfaces and in the matrix^36^. Biofilm appeared as fragmented pieces of coating on the textile fibers on both sides (Fig. 1b,c). Using doctor blading, we were able to control and manipulate the thickness of wet biofilm coating with 0.17 mm, 0.51 mm, and 1 mm masks (DB-0.17 mm, DB-0.51 mm, DB-1 mm). Such deposition resulted in a glossy, uniform, and thick layer of biofilm covering the front surface of the textile, with a decreased thickness after drying due to the loss of water in the cells, whereas little biofilm was seen on the back side (Fig. 1b,c). Finally, we used vacuum filtration (VF) to increase the interaction between the biofilm and the textile substrate^37,38^. We have previously employed this technique in a scalable protocol for purifying engineered curli fibers and integrating curli fusion proteins into non-woven textiles^38,39^. The optical microscope and scanning electron microscopy (SEM) images showed that the biofilm consisting of aggregated curli and cells was entrapped in the porous textile matrix, subsequently preventing more biofilm from passing through the textile. This method created a smooth and dense biofilm packing on the top surface of textiles and left little biofilm observed on the back side (Fig. 1b,c).

To quantify and compare the amount of biofilm integrated into the dry composites made with each approach, we measured the “integration density” of the biofilm-textile composites, which we defined as the dry weight of biofilm incorporated into per unit area of the composites. The results suggested that the three methods we employed resulted in the integration of 5–32 mg of biofilm per square centimeter of textile (Fig. 1d). An increasing amount of biofilm was distributed on both sides of the biofilm-adsorbed composites as the textiles were incubated in more concentrated biofilm solutions (Fig. S3). Doctor blading the textiles with biofilm pellets using masks of increasing thicknesses created thicker layers of coating only on the front side, leading to the highest integration density values (Fig. S3). We used the clogging of the textile and filter membrane as a sign to end the vacuum filtration process and did not further alter the integration density. The vacuum-filtered composites had an integration density greater than all the composites made with biofilm adsorption, suggesting the vacuum-aided filtration process rapidly accumulated more biofilm on the collecting textiles than the biofilm adsorption process in solutions. The wide range of integration density and the different morphological features have the potential to suit a variety of applications in the textile industry, ranging from functional apparel to technical textiles used in agriculture, construction, and other fields.

### Modified or improved physical and mechanical properties

The motivation to fabricate composites is to combine the advantage of each component^41^, so we investigated how biofilm integration modified the textiles’ characteristics. Specifically, we examined the biofilm-textile composites for performances of interest for wearable applications: surface wettability, breathability, and mechanical properties. Knowing that the composite morphology and the integration density can be altered by adopting different integration methods, we further assessed if these differences also impacted the composites’ physical and mechanical properties.

**Figure 2:**
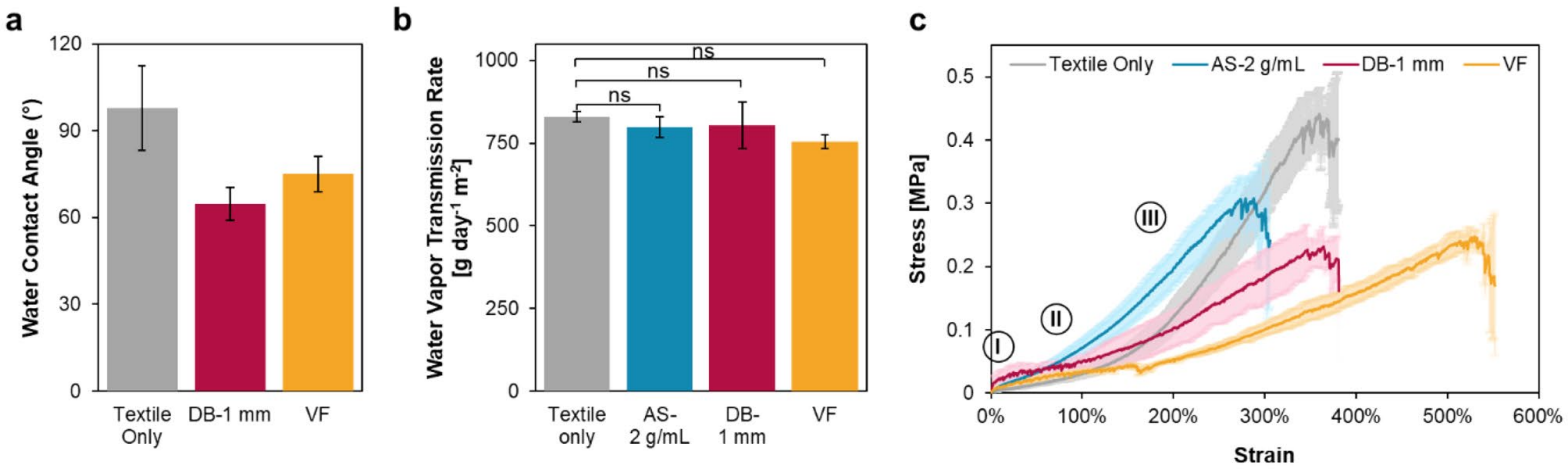
Effect of fabrication method on the physical and mechanical characteristics of biofilm-textile composites. (**a**) Bar plot of the water contact angle of the plain textile and composites revealing the modified surface wettability after the biofilm coating was introduced. The bars represent mean values, and the error bars are standard deviations. (**b**) Bar plot of the WVTR of the plain textile and the composites with the highest integration densities showing retained breathability after integration. The bars represent mean values, and the error bars are standard deviations. (**c**) Stress–strain curves of the plain textile and composites showing an improved Young’s modulus with adsorption (seen in Stage II) and elongation at break with vacuum filtration (seen in Stage III).

The moisture management properties of textiles, reflected by the textiles’ ability to remove sweat from skin, are essential to provide wearers with a satisfying level of thermo-physiological comfort in wearables^42^. As the first key step in the transportation of liquid sweat, wetting is determined by the textiles’ surface characteristics^43^. We studied how adding biofilm coatings using the three methods influenced the textiles’ surface properties by comparing the static water contact angles on the front surface of the composites with high densities of integration (AS-2 g/mL, DB-1 mm, VF). Both the doctor-bladed and the vacuum-filtered composites exhibited a decrease in the water contact angle (compared with the plain textile without biofilm integration) (Fig. 2a). In these composites, smooth biofilm layers covered the textile surface and reduced the macroscopic surface roughness that was caused by the textiles’ knitted structure and the cotton fibers protruding from the surface. The increased wettability of the doctor-bladed and the vacuum-filtered composites also agrees with the previously reported observation for curli thin films (contact angle of 58.6° ± 1.7°)^44^. We failed to record contact angle measurements with AS-2 g/mL, as the water droplet was absorbed immediately by the composites upon contact. As discussed in the previous section, unlike in the other two methods, the adsorbed biofilm did not completely fill up the pores on the textiles or cover all the textiles’ surface features. Compared to the plain textiles (porosity of 9%), we observed that the porosity of biofilm-adsorbed textiles (AS-2 g/mL) decreased to 3%. Adsorption might have modified the textiles’ pore size, surface roughness, and hydrophilicity to the extent that the capillary pressure was elevated, and the water droplet quickly filled the surface cavities^45,46^.

When sweat is transported in its vapor form by diffusion, adequate vapor transmission through the biofilm-textile composites must be ensured to establish a comfortable microclimate in wearing applications. We measured the water vapor transmission rate (WVTR) for composites with the highest integration densities prepared by each of the three methods (AS-2 g/mL, DB-1 mm, and VF), as we expected denser biofilm coating would hinder the transmission rate more than low-density coatings. Despite the reduced porosity, increased thickness, and the different moisture absorption capability of biofilm, we did not observe a statistically significant difference between the WVTR of each composite and that of the plain textile^47^ (Fig. 2b). Given that the WVTR of normal skin is 150–450 g m^−2^ day^−1^, the fabricated biofilm-textile composites remained breathable^48^.

Being mechanically strong is crucial for clothing fabrics to withstand daily wear and have a long lifetime, so we conducted tensile tests on the textiles before and after biofilm integration to determine the Young’s moduli and the elongation at break of the materials. Given that textiles’ mechanical behaviors vary with the direction of stretching in tensile tests, we performed all the mechanical tests in this study in the course direction perpendicular to the textiles’ grainline, as jersey fabrics are more elastic in this direction than in the direction of wales^49,50^. The stress–strain curves illustrated the distinctive mechanical responses displayed by the composites prepared with different fabrication methods (Fig. 2c).

We characterized the stress–strain responses of the textile and textile composites by stages (Fig. S4). In Stage I, crimp yarns of plain cotton textiles were first straightened before the textiles were extended, and the modulus increased. However, this response was not seen in the composites’ curves, which might be because of the presence of the biofilm that increased the friction between the yarns and restrained the stitch loops from changing their shapes. Stage II showed different mechanical behaviors of the tested materials: it was the linear elastic stage with reversible deformation caused by yarns moving within the textile structure for the plain textiles and AS-2 g/mL^49^, and the multi-cracking stage for DB-1 mm and VF. By calculating the Young’s moduli using the curve in this stage, we found that compared with the plain textiles (~ 33 kPa), biofilm adsorption increased the modulus by an average of 120% (~ 74 kPa) (Fig. S5). The porous structure of conventional knitted textiles formed by loops can effectively dissipate strain, rendering the textiles extensible and formable substrates in textile-based composites^51,52^. Dry biofilm, on the other hand, is stiffer and more brittle because of curli fibers’ rigid structures^44,53,54^. The biofilm coating might have strengthened individual yarns, reduced the pore size, and created yarn-to-yarn adhesion. Hence, as the integration density of the adsorbed biofilm increased with more concentrated biofilm solutions used, the overall strength of the composites was improved (Fig. S6). The stress–strain curves of DB-1 mm and VF showed minimal elastic deformation responses up to approximately 10% strain. In contrast, we observed repeated sudden stress drops and rises, a typical behavior of composites in which textiles are added to improve the mechanical performance and durability of inorganic matrices (e.g., textile-reinforced mortar)^55^. Each “spike” in the response represented a crack developed under load on the specimen, and more specifically, on the biofilm coating. As stress grew, crack propagation was also associated with large strain increases. Although the curves of DB-1 mm and VF had similar shapes in this stage, we noticed that the curve of VF spanned a wider range of strain, indicating that the biofilm was more well-integrated in the textile with vacuum-aided filtration than with doctor blading^56^. In Stage III, all the specimens underwent nonreversible deformation until failure. The plain textiles demonstrated an excellent elongation at break of 400%, which is a result of both the elastic spandex contained in the yarns and the knitted structure of the textiles^57^. Although we expected introducing biofilm to textiles would decrease the elongation due to the same yarn-restraining effect mentioned earlier, vacuum filtration, unlike the other two methods, improved the elongation at break of plain textiles by approximately 140%. Looking at the cross-section of the vacuum-filtered composites, biofilms were found to be deposited into the pores of the textile matrices. After drying, the loss of water in the “filler” biofilm caused the entire textile substrate to shrink. This shrinkage in the material would explain the overall increase in the percentage of elongation when the specimen broke, as it was compared to the initial shrunk state (Fig. S7).

### Water-induced self-repairing in biofilm-textile composites

Given that curli and curli-based composites films have been shown to intrinsically self-heal physical damages and recover functionalities after hydration^28,44^, we investigated whether integrating curli biofilm can facilitate repairs of macro-sized damages on textiles and enable the composites to regain their mechanical integrity and strength. Here, we employed two types of repairs commonly used for polymeric materials: patching and welding.

#### Self-repairing by patching

**Figure 3:**
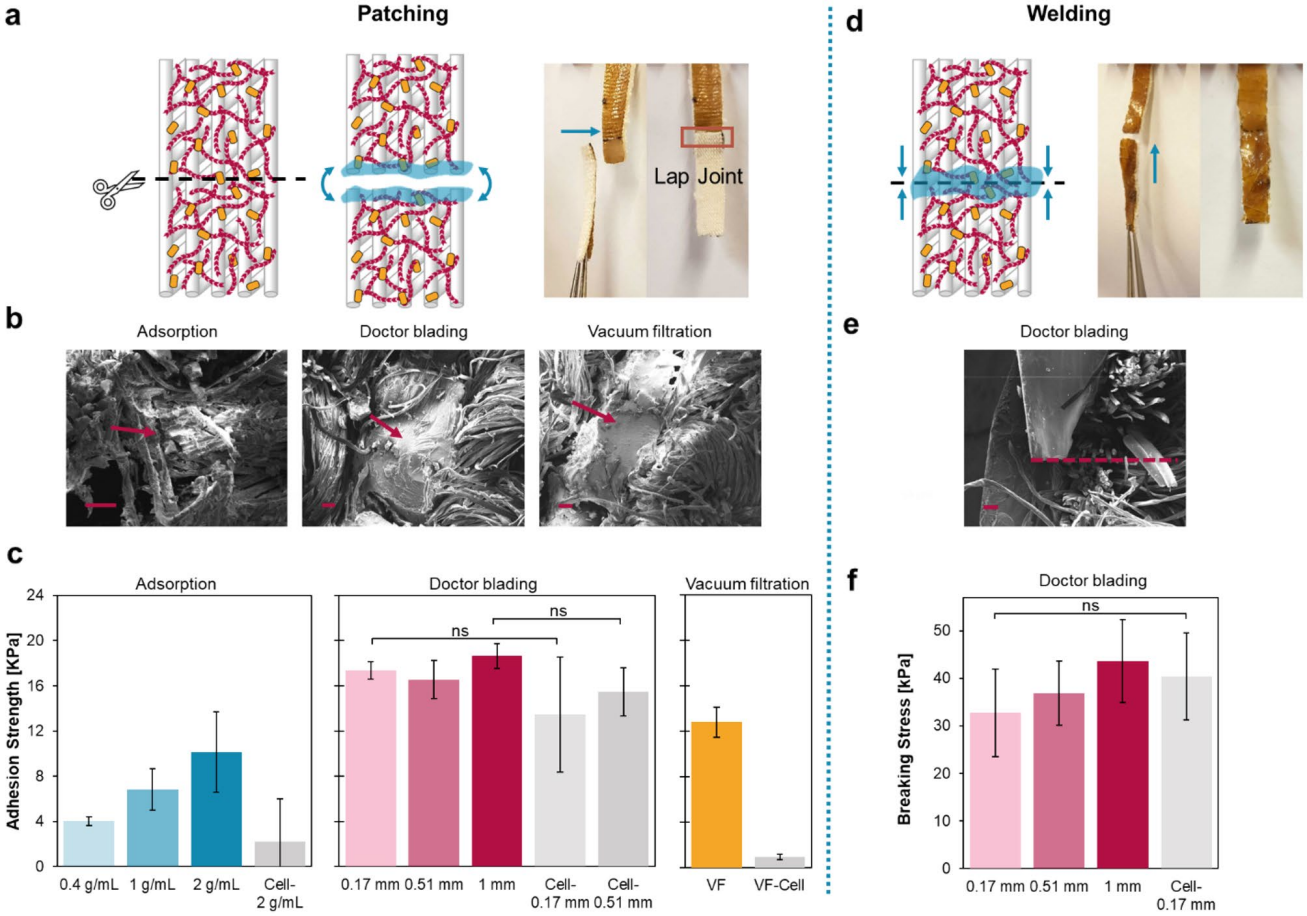
Self-repairing ability of biofilm-textile composites. (**a**) A schematic and an image showing the steps of using biofilm-textile composites as adhesive patches to repair cuts. (**b**) SEM images showing that the biofilm reformed in the overlap region joined the two composite pieces. The scale bars are 50 µm. The arrows point at the self-healed biofilm in the lap joints. (**c**) Adhesion strength obtained from the lap shear tests of the biofilm-textile composites and untransformed cell-only composites repaired by the patching method. The bars represent mean values, and the error bars are standard deviations. (**d**) A schematic and an image showing the cut repair by welding ends of two biofilm-textile composite pieces. (**e**) An SEM image of the cross-sectional view of a welded DB-1 mm composite with the textile substrate on the right and the biofilm layer on the left. The red dashed line indicates the cut made with scissors, which was repaired after the two composite pieces were welded by the cohesion of the biofilm layer. The scale bar is 50 µm. (**f**) Breaking stress of the composites doctor-bladed with biofilm and untransformed cells (DB-Cell-0.17 mm) obtained from the stress–strain curves. The bars represent mean values, and the error bars are standard deviations.

Patching refers to the process of covering broken materials with new materials or superficial patches^58^. Using native textiles as patches is preferred when repairing textiles in order to restore their original features. We tested the biofilm-textile composites as adhesive patches by cutting 1 cm wide composite strips into two pieces, rehydrating the two ends each with 20 µL of water, and overlapping the ends (with an area of 4 mm × 1 cm) (Fig. 3a). After drying, the two pieces of the composite were visibly joined into one piece by the overlap region. To visualize the repair mechanism in the overlap region, we imaged its cross section with SEM (Fig. 3b). We noticed that the biofilm coating from the two composite pieces healed and held the two pieces together. Specifically, in the adsorption method, the biofilm surrounding individual textile fibers held the yarns closely to each other; whereas in the other two methods, the cross-section images of the overlap region revealed that the biofilm layers self-healed into one thick layer in between the two composite pieces. This observation was the result of the differences in morphological and integration density stemming from the different integration methods.

To study the performance of the biofilm-textile composites as adhesive patches for repairing damages, we measured the adhesion strength of the composites fabricated by all three methods using a modified version of single lap shear tests (Fig. 3c). In traditional lap shear tests, shear forces are applied to separate two adherends that are glued by an adhesive, mimicking a typical failure mode of adhesive joints^59^. In our modified version, the adhesive is the biofilm that has already been integrated throughout each textile piece, not just in the overlap region; and the gluing relies on the self-healing of biofilm. The adhesion strength, defined as the ratio of the maximum load at fracture and the overlap area, was calculated for all the biofilm-textile composites with various integration densities as well as textiles integrated with untransformed bacteria cells made with the same fabrication methods (labeled as AS-Cell, DB-Cell, and VF-Cell). As shown in Fig. 3c, overall, the composites with high integration densities reported stronger adhesion. In the overlap region, when a higher density of curli fibers was hydrated and put into contact, more molecular interactions and fiber entanglement were created, which led to healing between the biofilms and the composites^28^. The rise in adhesion strength with increasing integration density of biofilm also agrees with Kendall’s mathematical model of crack propagation on lap joint, in which the maximum load is dependent on the elastic modulus and thickness of the adherends and the adhesive energy of the bond^60^. Looking at each fabrication method, we found the adhesion strength of the composites made with biofilm adsorption increased with the integration density when higher biofilm concentration was used, while the doctor-bladed samples with different thicknesses demonstrated adhesion strength comparable to each other. This observation indicated that doctor-bladed biofilm displayed comparable binding quality to the textile substrate independent of the biofilm load.

When examining the biofilm-textile composites (containing both cells and curli fibers) with textiles integrated with untransformed cells (no curli fiber expression), we first noticed low adhesion strength for AS-Cell-2 g/mL and VF-Cell (1–2 kPa) compared to the composites integrated with biofilm (4–13 kPa). Although incubating the textiles in 2 g/mL untransformed cell solution resulted in an integration density (~ 16 mg/cm^2^) comparable to AS-2 g/mL (14 mg/cm^2^), only one out of three specimens tested had its pieces joined. Considering that untransformed cells do not produce curli fibers, this observation indicated the pivotal role of curli fibers in biofilm self-healing. Also, since the untransformed cells were not entangled within curli fiber aggregates as extracellular matrix, they were more likely to penetrate the textile matrix than being adsorbed on the surface, thus unable to generate strong adhesion on the surface. Similarly, when vacuum filtering untransformed bacteria cells, the majority of the cells went through the textile directly and ended up in the filtrate, resulting in a substantially lower integration density (15 mg/cm^2^, compared with 25 mg/cm^2^ for VF), limiting the adhesion strength.

Contrastingly, the textiles doctor-bladed with the cells (DB-Cell-0.17 mm, DB-Cell-1 mm) displayed adhesion strength values without statistically significant differences with their biofilm-textile composite counterparts. The cell strain we used was derived from MC4100 (LSR10) and does not produce other common extracellular materials apart from curli fibers, such as fimbriae, flagella, cellulose, and lipopolysaccharide O antigen, which have been reported to contribute to the adhesion and/or cohesion of bacterial biofilm^34,61–65^. To understand the reason behind the high adhesion strength of samples prepared by doctor blading untransformed cells, we first calculated the samples’ integration density and noticed greater values compared with the biofilm-textile composite counterparts (containing both cells and curli fibers) with the same wet thickness. DB-Cell-1 mm achieved a 1.4-fold integration density compared to DB-1 mm, meaning the dry cell layer of the composites may contain a much greater number of cells than the biofilm layer (Fig. S8). A recent work making bulk stiff living materials with only PQN4 cells revealed the tight packing of the cells after casting and drying^66^. Furthermore, the characterization of the cast materials in this work suggested that the mechanical integrity was possibly contributed by the materials’ heterogeneity resulting from the intracellular components (e.g., nucleic acids, lipids, and proteins) released after cell death^66^. In our case, these compounds might have contributed to cell layers binding to the textile surface and covering the fibers, and to the cohesion reformed between the cell layers of patched composites containing doctor-bladed cells. In addition, the packing and the heterogeneity together allowed the dry-cast cells to have high Young's moduli ranging from 5 to 42 GPa, which further aided in the mechanical strength of the composites with doctor-bladed untransformed cells and their high adhesion strength^60,66^.

#### Self-repairing by welding

Although the adhesive biofilm-textile composite patches provide a simple repairing method and reliable outcomes, closing cuts directly on textiles without overlapping the ends is preferable, particularly for retaining aesthetic features and wearing comfort. Therefore, we explored welding as a second repair method, where we placed the ends of 1 cm wide textile strips that were cut into two together, rehydrated the ends with 40 µL of DI water, and dried the composites until the edges were fused (Fig. 3d). The self-healing of biofilm was more challenging with welding than with patching, as the cross-sectional area of the composites (< 0.1 cm^2^) was much smaller than the surface area of lap joints (0.4 cm^2^). Therefore, we chose to perform welding and the subsequent mechanical characterization only with the doctor-bladed composites that had the largest cross-sectional area and displayed the highest adhesion strength with the patching method.

All the doctor-bladed biofilm composites were able to fully close the cut through the cohesion of the biofilm layer on the textile’s front surface (Fig. 3e). To characterize the mechanical strength of the welded composites, we applied tensile loads on the healed cut and recorded the stress at breakage. In general, the composites tested broke at an average stress around ten times smaller than the breaking stress of plain textiles, because breaking the cotton yarns and fibers in plain textiles required large stress. All the doctor-bladed composites, including the untransformed cell-only composite control (DB-Cell-0.17 mm) had breaking stress values comparable with each other, demonstrating a similar quality of cohesion in the biofilm and cell layer (Fig. 3f). As explained in the previous section, the cohesion was likely a result of the interfibrillar and intermolecular interactions reformed in the biofilm layer and the dense packing and heterogeneous compounds in the cell layer.

**Figure 4:**
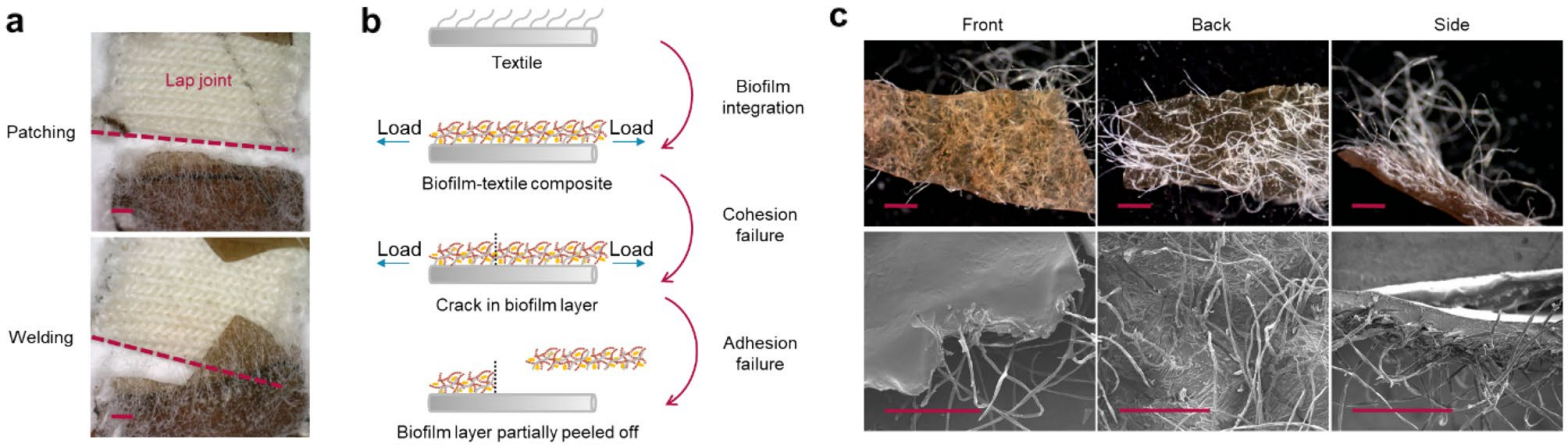
Mechanical failure of self-repaired composites. (**a**) Optical microscopy images of the patched and welded region of DB-1 mm composites that failed after the mechanical tests. The red dashes indicated the cuts made by scissors, repaired by patching and welding, and reformed after mechanical tests. Under the mechanical load, the biofilm layer from the top composite piece was partially peeled off together with the bottom composite piece from the textile substrate. (**b**) A schematic illustrating the mechanical failure of the biofilm-textile composites resulted from both the cracking in the biofilm layer (cohesion failure) and the detachment of the layer from the textile (adhesion failure). (**c**) SEM images showing the front, back, and side view of a biofilm piece peeled off from a doctor-bladed composite covering the textile fibers extruding from the textile surface.

When examining the composites after their mechanical failure, we noticed that the combination of adhesion and cohesion failure resulted in the mechanical failure in both patched and welded biofilm-textile composites (Fig. 4a). At the failure region, the biofilm layer was broken and detached from the textile. We summarized the failure mode in a schematic shown in Fig. 4b. In the lap shear test of a patched doctor-bladed composite, the specimen underwent a multi-cracking stage where cracks propagated from the two ends to the center (i.e., the lap joint). The final crack occurred at the edge of the lap joint, where the thickness of the biofilm layer changed due to the biofilm self-healing in the joint, and appeared straight, as shown in Fig. 4a (top). Then, the healed biofilm layer was peeled off from one of the two textile substrates under load, leading to an adhesion failure. A similar failure process happened when stress was applied to the welded composites, except that the last crack did not form at a consistent distance to the healed cut. Thus, the biofilm detached from the textile substrate did not have a consistent area, and the stress required to achieve such adhesion failure varied, as indicated by the irregular edge of the biofilm layer in Fig. 4a (bottom). To further understand the adhesion failure, we imaged a piece of biofilm layer peeled off from the doctor-bladed composites and saw that the textile fibers covered by the biofilm were also removed from the textile surface (Fig. 4c). These fibers protruding from the textile surface were embedded in the deposited biofilm layer, binding the biofilm firmly to the yarns. Therefore, the adhesion strength between the biofilm and the textile was impacted by the number of cotton fibers coated, in other words, the area of adhesion from the last crack to the cut. Since the composites prepared with doctor-bladed biofilm and those made with untransformed cells both created an even layer of coating over the same textile surface, it was likely that regardless of the layer’s thickness, their adhesion strengths were close.

### Challenges and outlook

In this study, we have constructed self-repairing curli-expressing biofilm-textile composites with adsorption, doctor blading, and vacuum filtration. These methods are fast and simple and can tune the morphological, physical, and mechanical characteristics of the resulting composites. Our results demonstrated that the composites can effectively self-repair visible cuts through patching and welding, and the strength of the restored sample resulted from the strong cohesion within the biofilm as well as the adhesion between biofilm coatings and textile substrates.

One challenge associated with depositing a biofilm layer on the surface of a soft textile substrate is biofilm buckling. When growing on dry substrates, biofilm forms three-dimensional buckles due to the internal mechanical stress created by the friction between the biofilm and the textile surface. The cohesion of biofilm promotes buckling while the biofilm-textile adhesion facilitates the stress to be transmitted to the textile substrate, initiating the deformation of the composites^67,68^. Providing external stress to the textile substrate can be a solution to reduce deformation. For instance, in this work, we flattened the integrated textiles before drying and kept the edges fixed by taping or clamping during the drying process to reduce wrinkles and buckles.

The biofilm-textile integration methods proposed in this study are deemed applicable in the clothing industry in the future because of the simplicity of fabrication, as well as the composites' retained breathability, enhanced mechanical strength, and effective water-inducible self-repairing ability. To implement such applications, however, we must face the difficulty posed by the long-term stability of biofilm coating during the routine use of biofilm-integrated clothing. For example, the bacteria in the biofilm are not expected to survive the detergents and rotation during laundry processes. A potential solution by Raab et al. suggested that dried bacteria or spores can be reloaded after laundry or before usage to regenerate biofilms^11^. Also, prior work on genetically engineered curli fibers vacuum-filtered onto non-woven textiles demonstrated the retention of curli on textiles when exposed to detergents, strong solvents, and constant agitation^38^. Therefore, an alternative solution to maintain the composites’ stability can be integrating purified and functionalized curli fibers without the biofilm on textiles to display self-healing and customizable features. We attempted self-repair after producing curli fibers modified to secreted extracellularly into the culture media, purifying the protein with a method based on vacuum filtration, and doctor blading the product protein hydrogel onto the textiles (0.17 mm wet thickness). The self-healing behavior of the curli adhered on the textiles enabled the patching repair of curli-textile strips, as demonstrated by applying loads on the tensile tester (see Supporting Information Movie S1), highlighting the involvement of curli fibers in the self-repair mechanism of biofilm-textile composites.

**Figure 5:**
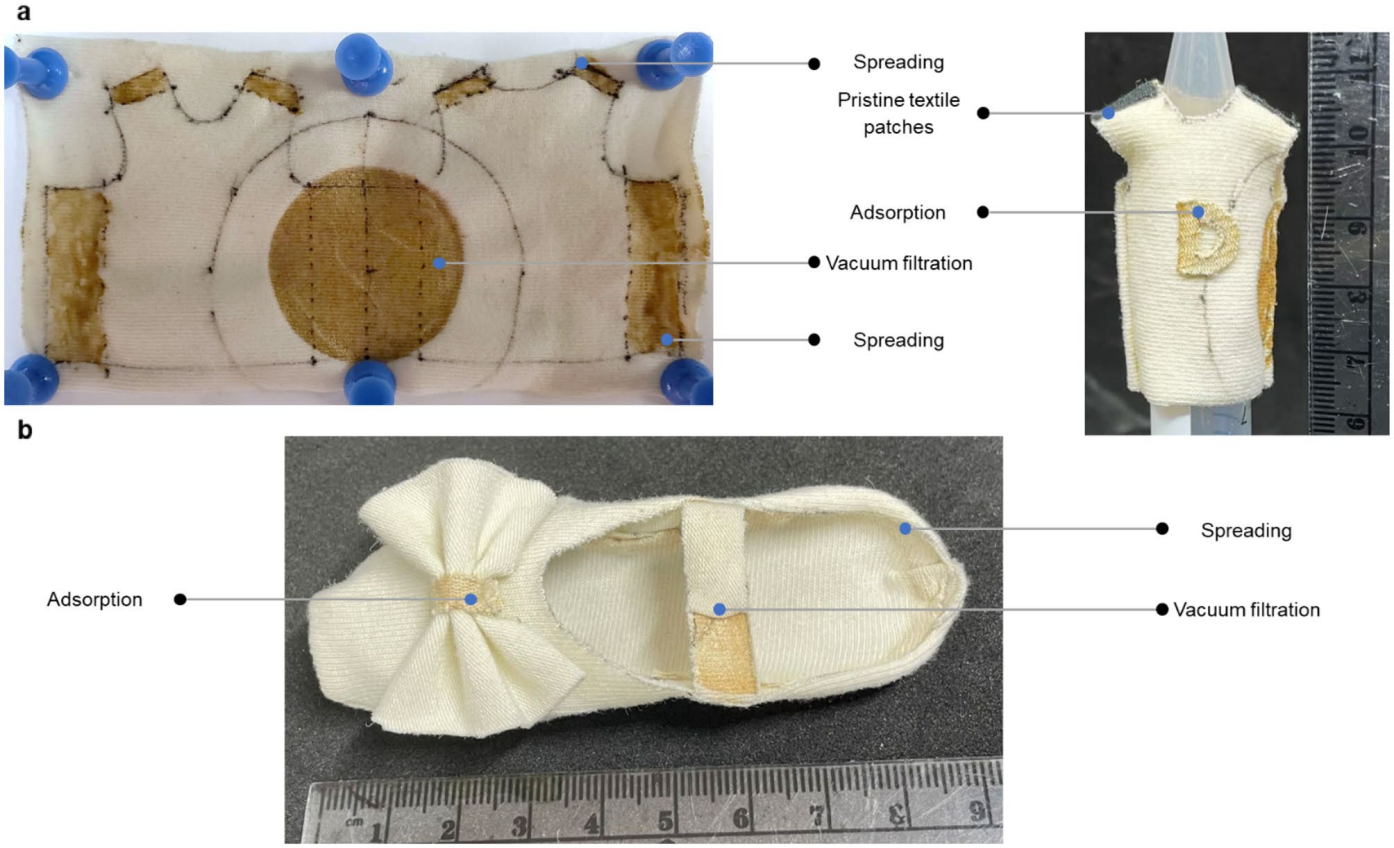
Garment prototypes fabricated based on the repair mechanism of biofilm-textile composites. (**a**) A mini shirt with an adhesive decoration patch made with biofilm-adsorbed composites in the front and shoulder pads made with regular plain textiles. (**b**) A mini dance shoe with an adjustable strap utilizing the patching repair of biofilm-textile composites.

To demonstrate the repair size of biofilm-textile composites and the ease of integration, we made two garment prototypes with textiles joined only by the integrated biofilm (Fig. 5). We replaced the doctor blading method with an even simpler spreading of the biofilm pellet over the designed overlap region with a spatula. The biofilm integrated with all three methods allowed for the adhesion initiated by rehydration between two pieces of composites. We have also demonstrated the possibility of adhering pristine textile to biofilm-textile composites using the patching method, by attaching two pristine textile patches to the shoulders of the mini shirt coated by biofilm. After being rehydrated and dried, the biofilm from the composite adhered irreversibly to the pristine textiles and joined the patches and the shirt together. These prototypes helped to reveal the potential of biofilm-based self-repair to replace sewing in making garments and maintaining their mechanical integrity.

Although we chose cotton-spandex knitted textiles in our demonstration, we expect that a wide selection of textiles can be used for fabricating biofilm-textile composites, and the resulting composites’ properties are affected by several characteristics of the textile material. Textiles with a moderate hydrophobicity and higher surface nanoroughness promote bacterial adhesion^69^, which may result in a higher integration density in composites with adsorbed biofilms, thereby augmenting the Young’s modulus and the self-repairing strength of the material. When the biofilm is vacuum-filtered or doctor-bladed onto the textiles, textiles with more fibers protruding from the surface could lead to stronger binding between the biofilm coating and the textile, thus improving the composites’ self-repairing performance in lap shear tests. Textile structures, such as woven, knit, and non-woven, are expected to also have an impact on the composites’ mechanical properties due to the different interactions of yarns under tensile loads. Textiles with smaller pore sizes entrap less biofilm in the textile matrix via vacuum filtration, which reduces the integration density of biofilm and the composites’ elongation-to-break. Therefore, the material of the textile substrate could be chosen to generate composites with application-specific properties.

The benefits of integrating bacteria biofilm can be maximized by utilizing the living properties of biofilm and displaying novel features through the material. SEM images of the biofilm composites prior to drying revealed that the textile fibers were covered with biofilm-containing bacteria cells (Fig. S9). Although some cells might have died during the composite fabrication, we expect the biofilm’s living properties can be restored to some extent after a more suitable living condition is provided. In fact, stiff materials made by casting and drying living bacteria cells have demonstrated the preservation of cell viability^66^. Using synthetic biology, these cells could be further engineered to tune the characteristics and adhesion of the biofilm, detect and respond to environmental stimulations, and display tailored properties to a broad range of applications. We envision the facile integration of biofilm has the potential to transform the conventional use of textiles, including clothing, household textiles, and industrial fabrics. The biofilm-textile composites will not only make these objects more durable and repairable under daily wear, but also smarter for their applications through the tunable biological properties of biofilms (self-regenerating, sensing, and responding to the environment). Combining the self-healing ability and the living properties, the biofilm-textile composites hold promise for driving the transition towards sustainable production and consumption of textiles, and even defining a multitude of future textile applications in unforeseen domains, such as art, medicine, and engineering.

## Methods

### Cell strains, plasmids, and curli expression

The pET21d-*csgBACEFG* plasmid encoding the entire curli operon, and the *E. coli* mutant strain, PQN4, from which the native curli operon has been deleted were gifted to us by the Joshi Lab (Harvard University, Boston, MA). This plasmid contains a T7 promotor for inducible curli expression, a glycine-serine-rich flexible linker (GSG)_4_, and a six-histidine tag at the C-terminus of CsgA to enable future immunodetection or purification. As a negative control for amyloid expression, a pET21d plasmid containing the *malE* gene encoding the maltose binding protein MBP) was used.

To express the protein, we first transformed the modified plasmid into electrocompetent PQN4 cells, which were then streaked onto a lysogeny broth (LB) agar plate containing 100 µg/mL carbenicillin and 0.5% (m/v) glucose (for catabolite repression of T7 RNA polymerase). As a control, we also streaked untransformed PQN4 cells onto an LB agar plate containing 25 µg/mL chloramphenicol. After overnight incubation at 37 °C, one colony was picked from each agar plate and inoculated into a 5 mL LB starter culture containing 100 µg/mL carbenicillin and 2% (m/v) glucose for the transformed cells and 25 µg/mL chloramphenicol for the untransformed cells. The starter cultures were grown overnight in an incubator shaker at 225 rpm and 37 °C. The next day, the stationary phase culture of transformed cells was diluted 100-fold in fresh LB media containing 100 µg/mL carbenicillin and cultured at 37 °C and 225 rpm for approximately 24 h to allow for protein expression. The untransformed cells were also diluted 100-fold with LB media (25 µg/mL chloramphenicol) and grown under the same condition. Both cultures were stopped when the cells reached an optical density at 600 nm of ~ 4 and centrifuged for 25 min at 4000×*g* for biofilm collection in the pellets. Each 500 mL culture yielded ~ 3.0 g curli-expressing biofilm pellet or ~ 2.2 g untransformed *E. coli* cell pellet.

### Textiles

The textile samples used in this study (94% cotton and 6% spandex) were knitted jersey fabrics provided by lululemon athletica inc. Knitted textiles have different patterns on their two sides, generated by the stitches in the knitting process. For consistency, we defined the side showing the loops of stitches (usually referred to as “the right side” for knitted fabrics) as the “front” side of the textile and deposited biofilm on this side. The “back” side reported in this study is defined as the side where the ends of the loops can be seen (usually referred to as “the wrong side” for knitted fabrics).

### Fabrication of biofilm-textile composites

Three methods were used to fabricate composites with the collected biofilm and the textiles: adsorption, doctor blading, and vacuum filtration.

#### Adsorption of biofilm on textiles

The pelleted biofilm from the culture of transformed bacteria was weighed and diluted to 0.4 g/mL, 1 g/mL, and 2 g/mL with deionized (DI) water. The textile samples with a dimension of 6 cm × 6 cm were immersed in 4 mL of biofilm solutions overnight in small Petri dishes at room temperature to allow for biofilm adsorption to the textiles. The following day, the textile composites were air dried at room temperature overnight.

#### Doctor blading of biofilm on textiles

The biofilm pellet was doctor-bladed onto the front side of textiles samples using a glass slide as a blade and masks made with one layer of glass coverslip (0.17 mm thick), three layers of glass coverslips (0.51 mm thick), and one layer of glass slide (1 mm thick) coated with a layer of Teflon tape. The composites were then air-dried at room temperature overnight.

#### Vacuum filtration of biofilm on textiles

The textile samples were cut into 7 cm × 9 cm pieces and pre-wetted in LB medium. The biofilm pellet was resuspended in LB to 0.65 g/mL. We performed preliminary tests by varying the biofilm concentration and did not observe any direct relevance between the biofilm concentration and the final amount of biofilm integrated. Knitted textiles have large pore sizes compared to the size of bacteria. To increase the amount of biofilm trapped during vacuum filtration, a polypropylene/polyethylene separator depth filter with 10 µm pores (VWR) was placed under the textile, as we have previously shown that curli fibers formed aggregates and did not pass through filter membranes with such pore size in vacuum filtration^39^. The filtration was stopped until the textile and filter membrane were completely clogged, and no liquid could pass through. Approximately 7 mL biofilm solution with a biofilm concentration of 0.65 g/mL was filtered under vacuum, leaving a layer of biofilm that covered an area of 7.55 cm^2^ on the front side of the textiles. The textile composites were air-dried at room temperature overnight.

### Fabrication of cell-textile composites

The pellets from the untransformed bacterial culture were weighed and used to fabricate cell-textile composites using the same three methods described above. In the adsorption method, the biofilm concentration and volume were kept the same. With doctor blading, cell layers of the same thicknesses were made on the textile surface. Without the curli fibers, the untransformed cells did not aggregate, thus being filtered through the textiles and 10 µm-pore size filter membranes in vacuum filtration. Despite the fact that the textile-filter membrane was not clogged, the concentration of the cell solution and the filtration volume were controlled to be the same as in the fabrication of biofilm-textile composites.

### Scanning electron microscopy

Scanning electron microscopy (SEM) images were taken for the textile composites prepared with the aforementioned methods and the dry biofilm layer peeled off from the doctor-bladed composites. In particular, to visualize the structure of biofilm coated on the textiles, the composites were treated for 15 min each with 25%, 50%, 75%, and 100% v/v ethanol and dried in a critical point dryer (Leica EM CPD300). The rest of the composites and the peeled-off biofilm underwent air drying. All samples were sputter coated with platinum to a thickness of 5 nm (Leica ACE600). The microscopy was performed with a FEI Quanta 450 Environmental Scanning Electron Microscope (Field Electron and Ion Company).

### Integration density measurement

The dry weights of plain textile samples before biofilm integration (${W}_{t}$) and the biofilm-textile composites (${W}_{c}$) were measured with Equinox Analytical Balance (Adam Equipment) for each integration method. The integration density represents the weight of biofilm integrated per unit area of textile. A minimum of three replicates were measured for each method, and the average (in mg/cm^2^) was reported.

1 $$Integration \, Density =\frac{{W}_{c} - {W}_{t}}{Area}$$

### Water vapor transmission test

The water vapor transmission rate (WVTR) was evaluated following the water method of ASTM E96 standard. Three samples of 1 cm × 1 cm were cut from the plain textile and the air-dried biofilm-textile composites were prepared with each of the three methods described. Before the test, all specimens had been stored and conditioned under the experimental conditions (20 °C, 50% relative humidity) for at least 24 h. In the test, the specimens were first fixed onto the mouths of open glass vials (inner diameter of 8 mm) containing 1 mL DI water with parafilm surrounding and sealing the circumference. Then, the glass vials were incubated at room temperature (20 °C) at a relative humidity of ~ 50% for 24 h. Before and after the incubation, weight measurements were taken with Equinox Analytical Balance (Adam Equipment). Using these measurements, the WVTR was calculated following the formula below.

2 $$WVTR =\frac{{W}_{i }-{ W}_{f}}{Area \times Time}$$

where ${W}_{i}$ is the initial weight of the glass vial and ${W}_{f}$ is its final weight after the 24-h incubation.

### Tensile test

The uniaxial tensile test was performed according to the ASTM D5053-06 method, with slight modifications, using Shimadzu EZ Universal Tensile Tester (Shimadzu), which is equipped with a load cell of 500 N. Strips of 1 cm × 6 cm were cut from the dry biofilm-textile composites and the composites integrated with untransformed PQN4 cells (not curli-producing) made with the three integration methods as well as the plain textile (with no biofilm). The specimens were gripped onto the tensile tester with a gauge length of 2 cm and stretched at a constant speed of 30 mm/min in the direction perpendicular to the textiles’ grainline until breakage. The tensile test was also done for the welded doctor-bladed composites at a speed of 10 mm/min. For each type of composite, a minimum of three tests were carried out. The average stress–strain curves were obtained by averaging the calculated stress (MPa) corresponding to every 0.1% strain increment.

### Contact angle measurement

Contact angles were measured by OCA 15EC (Dataphysics) using DI water droplets of 1 µL on the front side of the plain textile and the biofilm-textile composites. The images were taken and analyzed by ImageJ, and the average contact angles of quadruplicates were calculated.

### Self-repair of composites and single lap shear test for composite patches

The biofilm-textile composites with a dimension of 1 cm × 6 cm were cut in the middle into two pieces using scissors. To heal the cut, the two pieces were joined either by aligning the ends (welding) or by overlapping the ends (patching). In the welding method, the two edges of the cut were placed right next to each other and rehydrated with 40 µL DI water in a petri dish. The composite strips were taped to the petri dish to ensure that the ends were held together while self-healing, and then air dried on the bench. In the patching method, the ends (with a dimension of 1 cm × 4 mm) of the composite pieces were wetted with 20 µL DI water each and then sticked together with the biofilm-coated side facing toward each other. While the composites were air dried on the bench, the two ends were kept in contact by placing a 25 mm × 75 mm glass microscope slide on the top of the overlap region.

To characterize the adhesion strength of the textile composite patches, which is calculated by dividing the maximum force applied by the adhesion area, single lap shear tests were carried out using Shimadzu EZ Universal Tensile Tester (Shimadzu). The self-repaired specimens were gripped, and the distance between the two grips was controlled to be 2 cm for all the tests. A shear rate of 30 mm/min was applied until the healed two pieces were pulled apart. Quadruplicates were done for each integration method, and the average adhesion strength values were calculated.

The same lap shear test was performed with the cell-textile composites made with the three methods using untransformed cells, except for the specimens that remained as two pieces after being dried (Adhesion Strength = 0 kPa).

3 $$Adhesion \, Strength=\frac{{F}_{max}}{Adhesion \, Area}$$

### Statistics

Paired t-tests were performed on SPSS Statistics (IBM) to compare (1) the WVTR of biofilm-textile composites made with each of the three methods with the rate of the plain textiles, (2) the Young’s modulus of plain textiles and AS-2 g/L composites, (3) the adhesion strength of patched DB-0.17 mm and DB-1 mm with the strength of composites made with untransformed cells, and (4) the breaking stress of welded DB-0.17 mm with that of the cell-textile composites. A probability (p-value) less than 0.05 was considered significant. *p* > 0.05 was denoted as “not significant” (ns).

### Fabrication of textile composites with purified curli fibers

Curli fibers were expressed with PQN4 cells and the same plasmid encoding curli operon with *csgB* gene deleted to enable curli fibers to be secreted into the medium. Then, the curli fibers were harvested in a hydrogel form from the medium following a vacuum filtration-based purification method we previously established^39^. The curli fibers were doctor-bladed onto the textile with a 0.17 mm thick mask and dried at room temperature overnight.

## Supplementary Information

Supplementary Figures.

Supplementary Movie 1.

## Data Availability

The data that support the findings of this study are available from the corresponding author upon reasonable request.
