# Supplementary material for: Endowing textiles with self-repairing ability through the fabrication of composites with a bacterial biofilm
Source: Sci Rep. 2023 Jul 14;13:11389. doi: 10.1038/s41598-023-38501-2 (PMC10349112; doi:10.1038/s41598-023-38501-2)
Supplement: Supplementary file 1 — Supplementary Figures. [file 41598_2023_38501_MOESM1_ESM.docx]

**Endowing textiles with self-repairing ability through the fabrication of composites with a bacterial biofilm**

*Anqi Cai, Zahra Abdali, Dalia Jane Saldanha, Masoud Aminzare, Noémie-Manuelle Dorval Courchesne**

Department of Chemical Engineering, McGill University, 3610 University Street,

Montreal, QC, Canada, H3A 0C5

*E-mail: noemie.dorvalcourchesne@mcgill.ca

**Supporting Information**


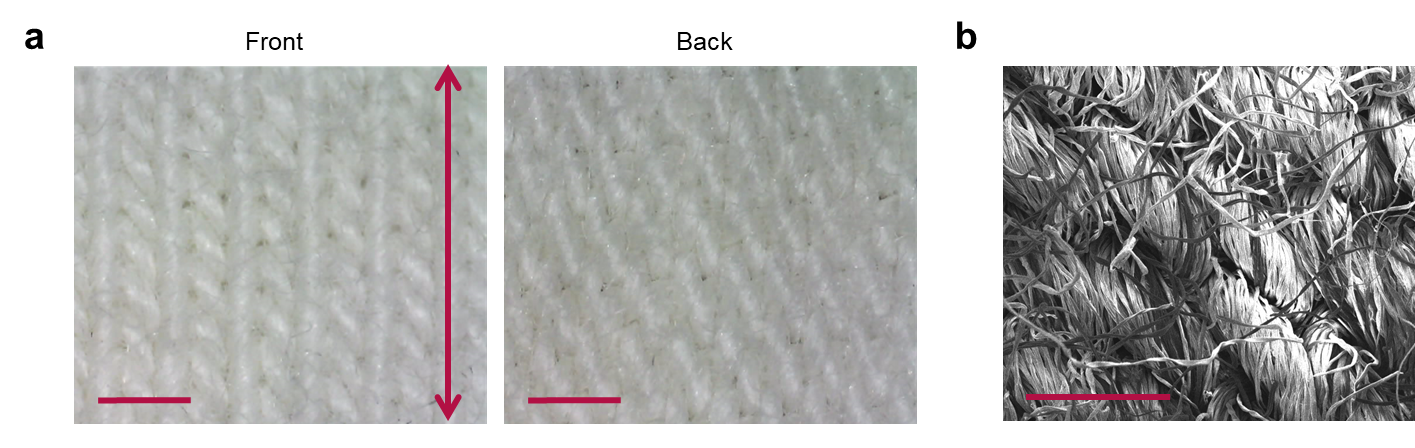


*Figure S1.* *The cotton-spandex knitted textile used as the substrate of the biofilm-textile composites. (a) Optical microscope images showing the knitted features of the front and back sides of the textile. The red arrow aligns with the grainline of the knitted textile, with the arrows pointing toward the wale direction. The scale bars are 1 mm. (b) An SEM image showing that the textile was knitted with yarns that have an average diameter of ~200 µm and consist of fibers ~16 µm in diameter. The scale bar is 500 µm.*


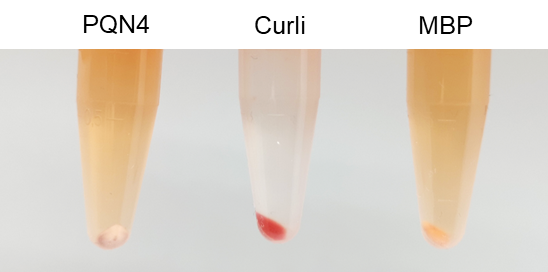


*Figure S2.* *Results of the Congo Red binding assay revealing that amyloid curli fibers have been produced in the biofilm and bound to Congo Red to form a red pellet. The cultures of untransformed PQN4 cells and the cells producing maltose binding protein (MBP) used as controls both showed white pellets and red supernatant.*

**
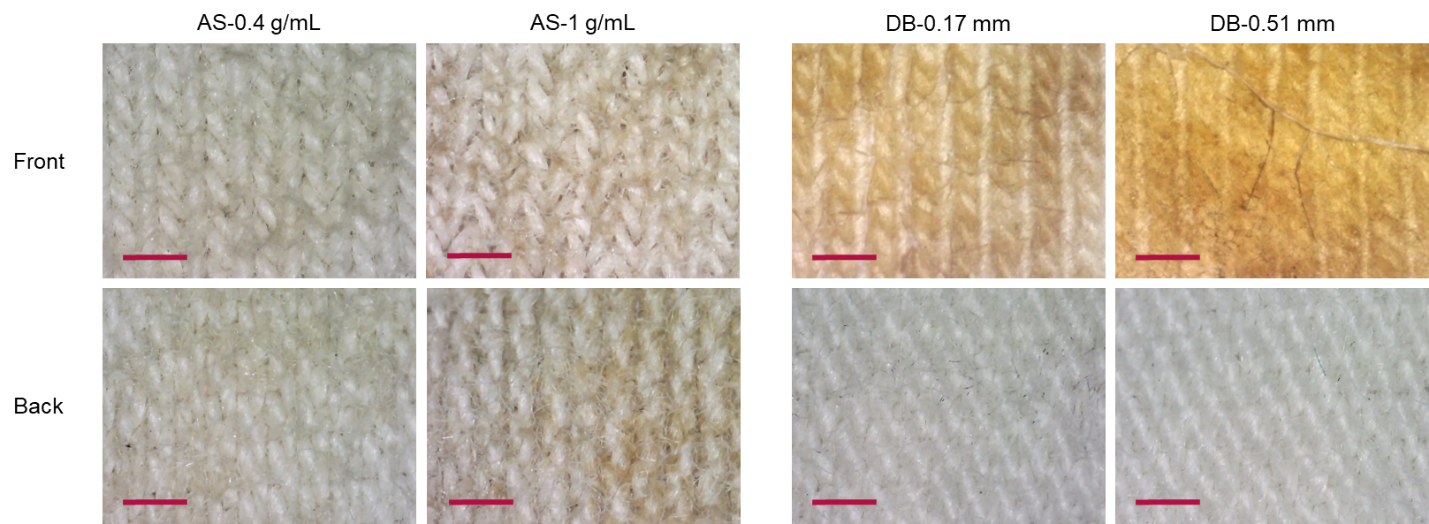
**

*Figure S3.* *Optical microscope images showing the biofilm distribution on the biofilm-textile composites made by varying the concentration of the biofilm solution (adsorption) and the thickness of the mask (doctor blading). The scale bars are 1 mm.*

**
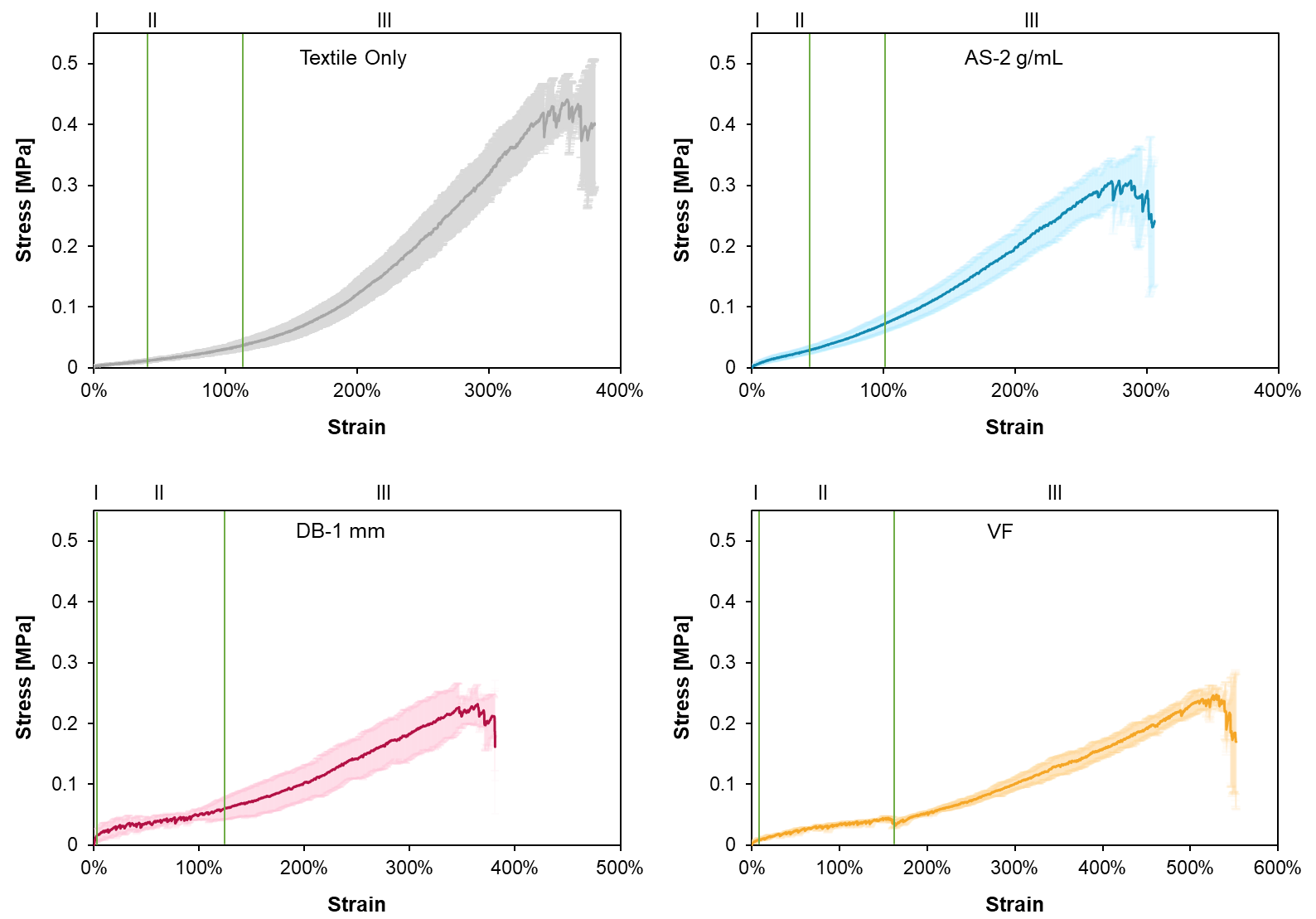
**

*Figure S4.* *The three stages of the stress-strain responses identified for the textile and the biofilm-textile composites.*


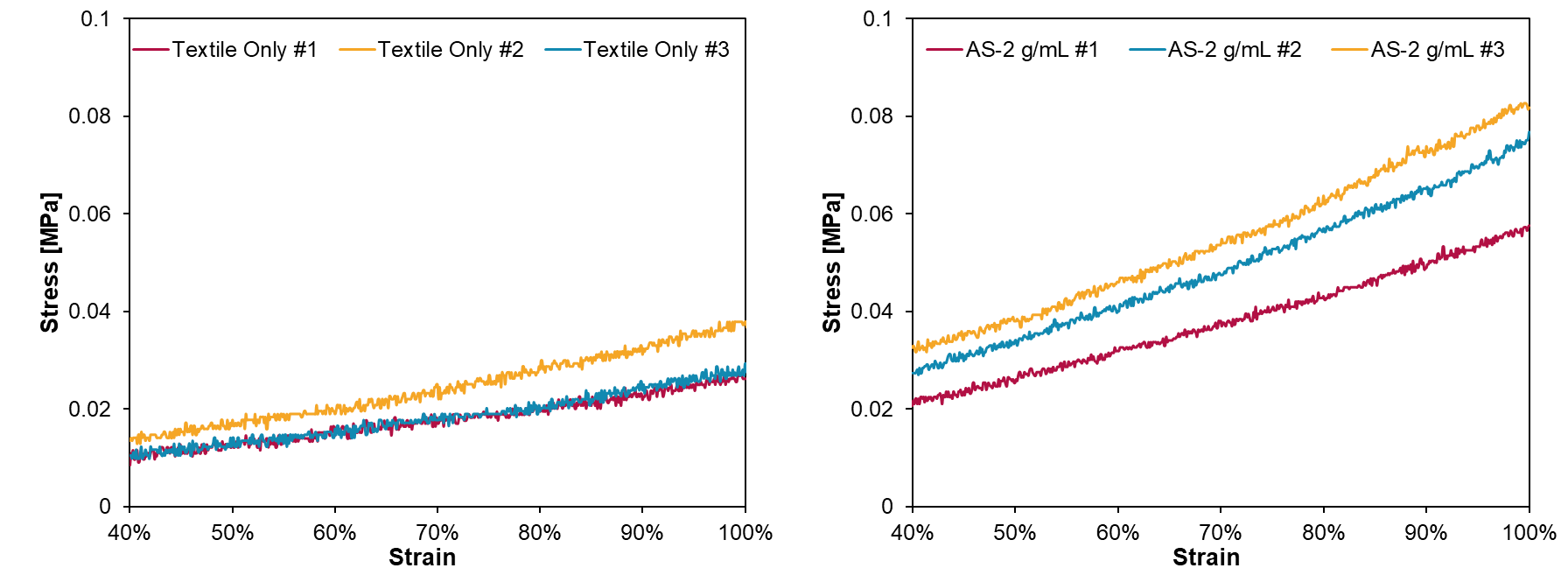


*Figure S5.* *The elastic stage (Stage II) of the stress-strain curves plotted for the triplicates of plain textiles and the AS-2 g/mL composites. The Young’s moduli of the specimens calculated based on this section of the curve suggested that the biofilm adsorption with 2 g/mL biofilm solution significantly improved the modulus of the textile (p<0.05).*

**
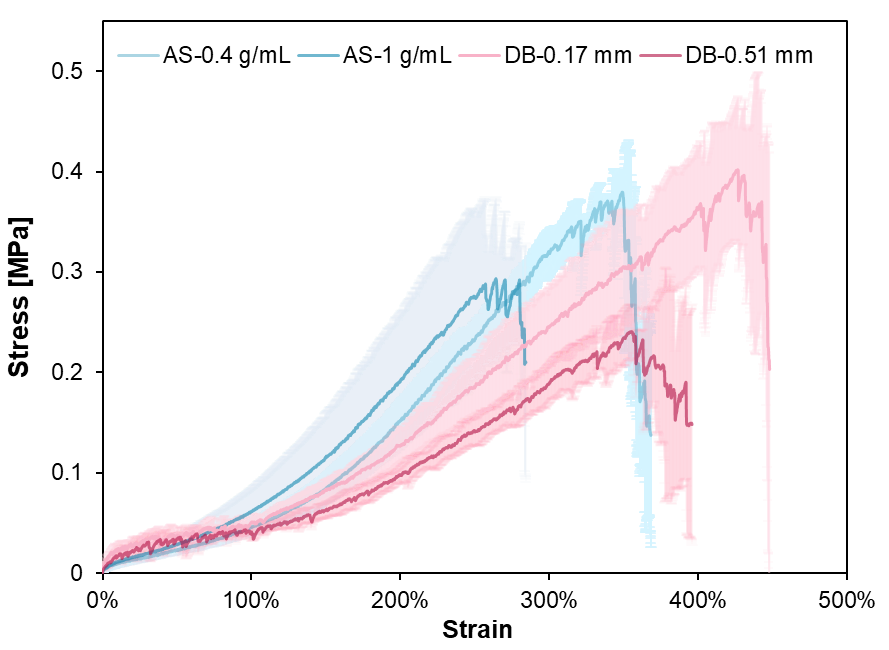
**

*Figure S6.* *Stress-strain curves illustrating the evolving mechanical responses (Young’s modulus, elongation at breakage) of the biofilm-textile composites as the integration density changed.*

**
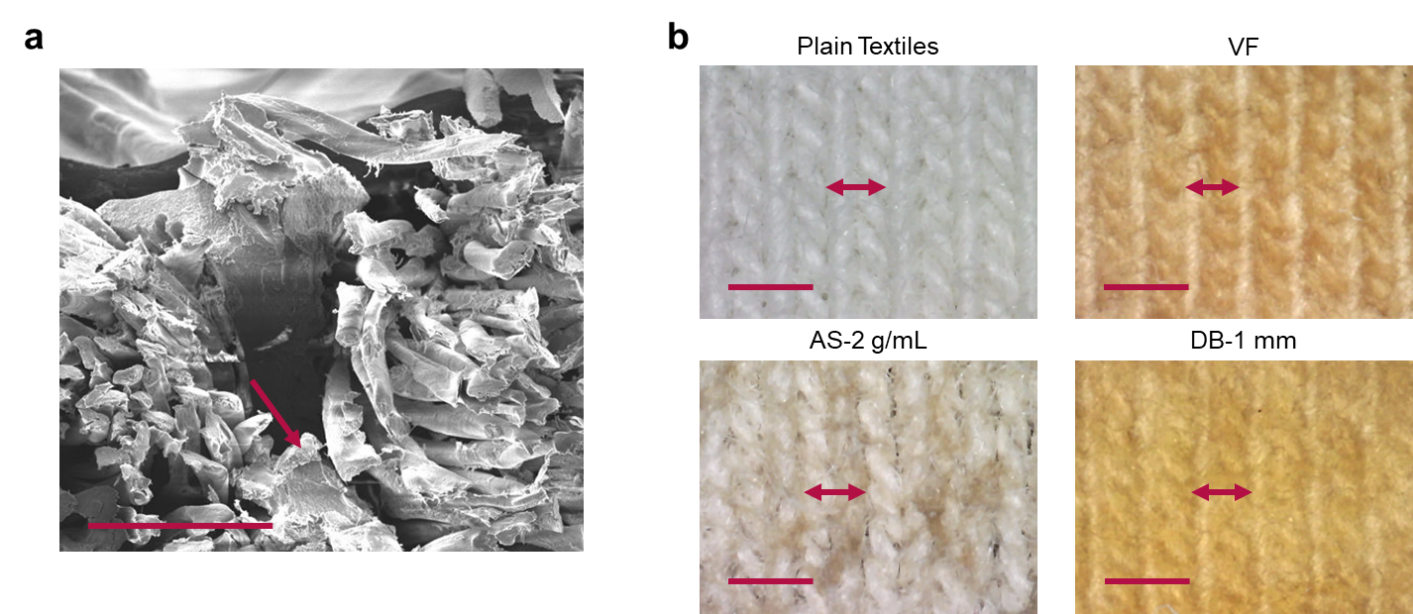
**

*Figure S7.* *Images revealing the impacts of vacuum-filtered biofilm on the elongation of break of the composites. (a) An SEM image of the cross-section of a VF composite showing the biofilm entrapped in the porous textile matrix. The red arrow points at a cluster of biofilm formed in between the textile fibers. The scale bar is 100 µm. (b) Optical microscope images demonstrating the shrinkage in size after the fabrication of the VF composite. The distance from the left edge of a grainline to that of the next grainline (indicated by the double-headed arrows) was reduced from ~0.75 mm to ~0.61 mm. The distance was measured by averaging the total distance spaced by six adjacent grainlines. As a comparison, the distance was ~0.77 mm for AS-2 g/mL and ~0.66 mm for DB-1 mm. The scale bars are 1 mm.*


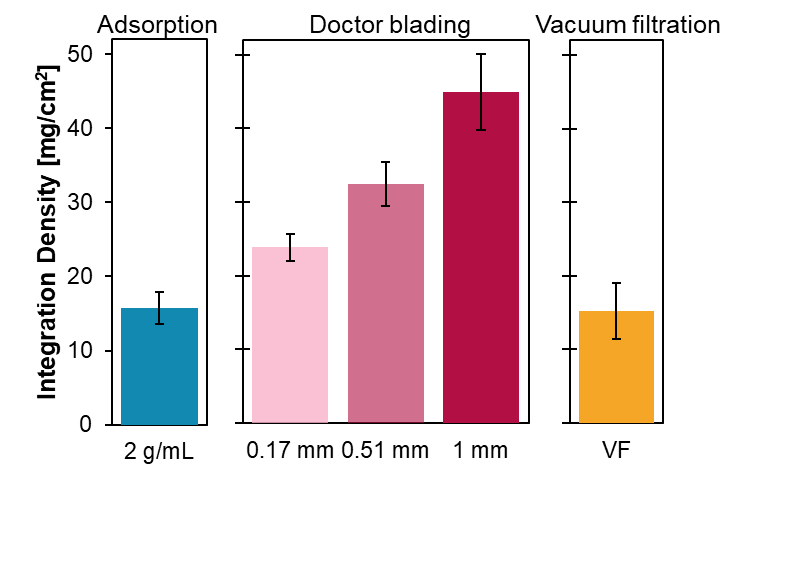


*Figure S8.* *Bar plot of integration density calculated for cell-textile composites prepared using the same three methods discussed and untransformed cells. The bars represent mean values, and the error bars are standard deviations.*

**
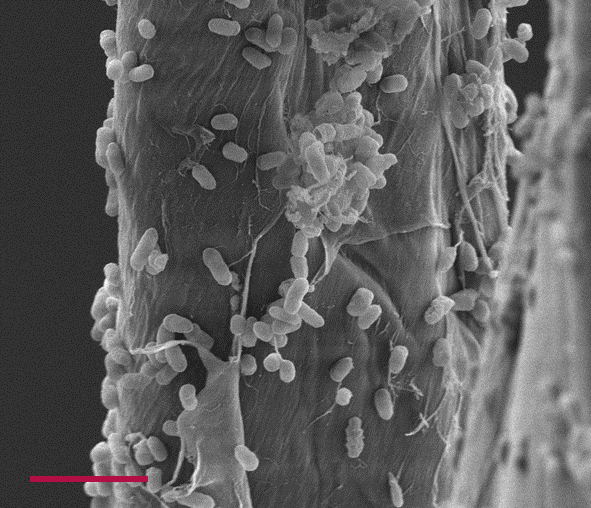
**

*Figure S9.* *An SEM image showing the bacteria cells and biofilm adsorbed on a single textile fiber. The scale bar is 5 µm.*

*Movie S1.* *A movie* *showing the self-repairing of textile patches doctor-bladed with purified curli fibers (integration density of 0.8 mg/cm^2^).*
